# Nutritional factors and survival in a cohort of patients with myelofibrosis

**DOI:** 10.3389/fnut.2025.1704844

**Published:** 2025-12-15

**Authors:** Silvio Buscemi, Piero Colombrita, Marco Santoro, Cristiana Randazzo, Carola Buscemi, Anna Maria Barile, Roberta Caruso, Martina Lombardo, Salvatrice Mancuso, Orazio Gambino, Giuseppe Bazan, Sergio Siragusa

**Affiliations:** 1 Department of Promozione della Salute, Materno-Infantile, Medicina Interna e Specialistica di Eccellenza (PROMISE), University of Palermo, Palermo, Italy; 2 Clinical Nutrition, Obesity and Metabolic Diseases Unit, University Hospital Policlinico “P. Giaccone”, Palermo, Italy; 3 Hematology Unit, University Hospital Policlinico “P. Giaccone”, Palermo, Italy; 4 Internal Medicine Unit, "V. Cervello" Hospital, Ospedali Riuniti "Villa Sofia-Cervello", Palermo, Italy; 5 Department of Engineering, University of Palermo, Palermo, Italy; 6 Department of Biological, Chemical and Pharmaceutical Sciences and Technologies (STEBICEF), University of Palermo, Palermo, Italy

**Keywords:** myelofibrosis, malnutrition, survival, phase angle, energy expenditure, splenomegaly

## Abstract

**Introduction:**

Myelofibrosis (MF) is classified among the chronic myeloproliferative neoplasms and presents unique nutritional challenges. Inflammation can trigger metabolic changes that lead to malnutrition and, ultimately, cachexia. Splenomegaly, which may occupy much of the abdomen and compress the stomach, can cause early satiety and further contribute to malnutrition. We investigated associations between nutritional parameters, clinical features, and survival in individuals with MF.

**Methods:**

Forty-five individuals with MF (21 males, 24 females) were included and compared with a healthy control group of 351 individuals (157 males, 194 females). Body composition (bioelectrical impedance analysis), resting metabolic rate (RMR), and substrate oxidation (indirect calorimetry) were assessed.

**Results:**

The mean follow-up was 31 ± 8 months, during which seven deaths occurred. MF was associated with malnutrition; patients exhibited lower bioelectrical phase angle values and higher RMR (32.4 ± 4.2 vs. 28.5 ± 3.6 kcal/kg-fat-free mass/24 h; *p* < 0.001) compared with controls. Kaplan–Meier survival curves showed that a phase angle (PA) below the median was associated with a lower survival rate (*p* < 0.005). Similarly, spleen length above the median was linked to poorer survival (*p* < 0.05).

**Discussion:**

Nutritional factors may serve as important predictors of survival in individuals with MF and should be considered in future supportive interventions aimed at improving both survival and quality of life.

## 1 Introduction

Myelofibrosis (MF) is a Philadelphia-negative chronic myeloproliferative neoplasm. It may present as primary myelofibrosis (PMF), which arises *de novo*, or as secondary MF, which develops from polycythemia vera or essential thrombocythemia, both included among the myeloproliferative neoplasms (1–3). The disease primarily affects older adults, with an annual incidence ranging from 0.22 to 0.99 cases per 100,000 and an overall incidence of 0.47 cases per 100,000 individuals per year (4). MF is characterized by elevated proinflammatory cytokines (TNFα, IL-6, IL-8, IL-12, IL-15, and IL-2R), which contribute to common symptoms such as night sweats and pruritus. This chronic inflammatory state induces metabolic alterations that can progress to malnutrition and eventually cachexia. Malnutrition significantly compromises the quality of life, the prognosis, and survival in individuals with oncohematologic disorders. A subanalysis of the COMFORT-I study (5) demonstrated that patients treated with Janus Kinase 2 (JAK2) inhibitors, agents that block various cytokines and hematopoietic growth factors, showed marked improvement in metabolic and nutritional parameters. Recent studies (6) have further highlighted correlations between nutritional indicators and prognosis in both PMF and secondary MF. Splenomegaly, a hallmark of MF, may extend to the left iliac fossa and occupy most of the abdominal cavity (7). This condition causes a sensation of heaviness in the left hypochondrium and, by compressing the stomach, leads to early satiety and increased risk of recurrent splenic infarctions. Hepatomegaly occurs in 40–70% of patients and may be complicated by portal hypertension (8), with clinical consequences such as ascites, esophageal and gastric varices, gastrointestinal bleeding, and portosystemic encephalopathy. In addition, the catabolic state of MF can result in hyperuricemia, predisposing patients to recurrent monoarticular or polyarticular gout.

Although MF presents distinctive features associated with malnutrition, no studies have directly evaluated the relationship between MF, nutritional status, and prognosis. In this study, we investigated possible associations between nutritional parameters and clinical characteristics in patients with primary or secondary MF.

## 2 Materials and methods

### 2.1 Participants

Patients diagnosed with primary or secondary MF at the Hematology Unit of the University Hospital Policlinico “P. Giaccone” in Palermo, Italy, were enrolled between January 2021 and December 2022. All nutritional evaluations were performed at study enrollment by a dedicated team of physicians and dietitians from the Unit of Clinical Nutrition, Obesity, and Metabolic Diseases, who also conducted follow-up visits. The present analysis includes only baseline nutritional measurements and survival data collected through October 2024. For comparison, the dataset was analyzed against a control group consisting of age- and sex-matched, non-diabetic, healthy individuals not currently using statins or other lipid-lowering agents. These controls were drawn from the database of the Nutrition, Cardiovascular Wellness and Diabetes (ABCD_2) project (ISRCTN15840340), a longitudinal single-center, observational study of a cohort representative of the general population of Palermo, the largest city in Sicily, as previously described (9). The ABCD_2 cohort was evaluated by the same Clinical Nutrition team between March and July 2015.

All MF patients met the World Health Organization (WHO) 2016 diagnostic criteria for primary MF (2) and the International Working Group for Myelofibrosis Research and Treatment (IWG-MRT) criteria for secondary MF (3). The degree of bone marrow fibrosis was assessed according to the current European consensus (10). Mutation status for JAK2, calreticulin (CALR), and myeloproliferative leukemia virus oncogene (MPL) was determined. The Dynamic International Prognostic Scoring System (DIPSS) (11) was used to estimate patient survival.

The institutional ethics committee (“Palermo 1” of the Policlinico “P. Giaccone” University Hospital, 25 November 2020, Ref: 10/2020) approved the study protocol, and all participants provided written informed consent. All procedures involving human participants were conducted in accordance with the Declaration of Helsinki and institutional guidelines.

### 2.2 Anthropometric and clinical measurements

Height and body weight were measured with participants lightly dressed and without shoes (Seca GmbH, Hamburg, Germany). Body mass index (BMI) was calculated as weight in kilograms divided by height in square meters. Fat mass (percentage of body weight) and fat-free mass (FFM) were estimated using bioelectrical impedance analysis (BIA) (12). An 800-mA, 50-kHz, tetrapolar impedance plethysmography (BIA-101 Anniversary, Akern Srl, Florence, Italy) was used to measure body resistance (R, ohm), reactance (Xc, ohm), and phase angle (PA, degrees calculated as arctan (Xc/R) × (180/*π*)). The use of crude BIA parameters, particularly PA, has gained attention as an indicator of intra- and extracellular hydration and nutritional status (13). Body circumferences were measured at the umbilicus (waist circumference) and at the most prominent buttock level (hip circumference), and the waist-to-hip ratio was calculated as an index of fat distribution. Grip strength was assessed with a hydraulic hand dynamometer (model SH5001; Jamar, Saehan, Republic of Korea). Participants performed the test while seated, with the shoulder adducted, elbow flexed to 90°, and forearm and wrist in a neutral position. They were instructed to exert maximal isometric contraction. Each hand was tested three times at 15–20-s intervals, and the average value (kg) was used for analysis (14). Systolic and diastolic blood pressures were measured twice at 5-min intervals in the seated position using standardized procedures (Omron M6; Omron Health Care Co., Matsusaka, Mie, Japan). Habitual dietary intake was assessed with a validated food frequency questionnaire (FFQ) developed for the local population (15). Participants reported their usual dietary habits over the past year, including meal patterns and food choices. FFQ data were analyzed to characterize overall dietary intake.

The mini nutritional assessment (MNA) was administered as previously described (16). An MNA score < 17 indicated malnutrition, and a score of 17–23 indicated risk of malnutrition.

In concomitance with baseline nutritional measurements, spleen length was measured with an ultrasound (Philips Epiq 5G; Bothell, WA, UA), a longitudinal view was taken during suspended respiration, placing calipers from the superior border (near the diaphragm) to the tip of the spleen. The same ultrasound examination described the presence or absence of hepatomegaly based on the measurement (data not reported in the report) of liver diameters.

Bone marrow biopsy was performed at diagnosis and repeated at the discretion of the hematological specialists in the event of clinical worsening of the disease. The data from the most recent biopsy were considered for the purposes of the study. Bone marrow fibrosis grading (2) was defined using Gomori’s silver impregnation.

### 2.3 Indirect calorimetry

Resting metabolic rate (RMR) was measured by indirect calorimetry using a ventilated hood system (Quark RMR; Cosmed, Rome, Italy), as described previously (17). The device was equipped with an infrared analyzer for carbon dioxide (VCO_2_) and a zirconium cell analyzer for oxygen (VO_2_). The analyzers were calibrated before each test with gases of known oxygen and carbon dioxide concentrations. Respiratory gas exchange was continuously measured for 1 h, and data from approximately 30 min of stable measurements were analyzed. The mean intrasubject variability for RMR was 3.9%. RMR was calculated using the Weir equation (18) and expressed in absolute terms (kcal/24 h) and relative to FFM (kcal/kg FFM/24 h).

### 2.4 Laboratory exams

Fasting blood samples were collected after an overnight fast of at least 8 h. Fasting plasma glucose (FPG), total cholesterol, high-density lipoprotein cholesterol (HDL-C), triglycerides (TG), uric acid, and creatinine were determined using standard clinical chemistry methods (Glucosio HK UV; Colesterolo tot. Mod P/D; Colesterolo HDL gen 3 mod P/917; Trigliceridi; Acido urico MOD P/917; Creatinina enzimatica; Roche Diagnostics; Monza, Italy). Serum low-density lipoprotein cholesterol (LDL-C) was calculated using the Friedewald formula, and insulin resistance was estimated using HOMA-IR, as described by Matthews et al. (19).

### 2.5 Statistical analysis

Continuous variables are presented as means ± SDs, and categorical variables as percentages. Student’s *t*-test was used to compare continuous variables between groups, and the *χ*^2^ test to compare categorical variables. Pearson correlation coefficients were calculated to examine associations among variables. Factors significantly correlated with PA were entered into a stepwise multivariate regression (forward model selection), with variables retained if they improved model fit (*F*-test, *p* < 0.15). All-cause mortality was the primary outcome. Kaplan–Meier survival curves were constructed, and differences were tested with the log-rank test. Referent categories were defined as the lowest median values of PA, spleen length, and MNA score, or by DIPSS categories and fibrosis grade. A two-tailed *p* < 0.05 was considered statistically significant. Analyses were performed with Systat software (version 13.0 for Windows, San Jose, CA, USA).

## 3 Results

In total, 45 (21 men and 24 women) individuals with MF and a duration of the disease of 72 ± 56 months were recruited; their clinical characteristics are presented in Table 1. Only one participant was using corticosteroids at the time of the first assessment.

**Table 1:** Clinical characteristics of patients with myelofibrosis.

	Patients with myelofibrosis
Diagnosis (*n*, %)	
Primary	33 (73.3)
Secondary	12 (26.6)
Bone marrow fibrosis (*n*, %)	
Grade 0-I	23 (51.1)
Grade II-III	22 (48.9)
JAK2 mutations	28 (62.2)
CALR mutations	6 (13.3)
MPL mutations	0 (0)
Treatment (*n*, %)	
Hydroxyurea	22 (48.9)
Ruxolitinib	12 (26.7)
Spleen longitudinal length (cm)	14.7 ± 3.9
Splenectomized (*n*, %)	2 (4.4)
Time from diagnosis (months)	72 ± 56
Follow-up of the study (months)	31 ± 8
Deaths at follow-up (*n*, %)	7 (15.6)
Circulatory blasts >1% (*n*, %)	7 (15.6)
White blood cells (× 10^9^/l)	11.6 ± 11.4
Hemoglobin (g/dL)	12.5 ± 2.3
Platelets (× 10^9^/L)	487 ± 296
Absolute lymphocyte count (× 10^9^/L)	2.22 ± 2.06
DIPSS risk category (*n*, %)	
Low	12 (26.7)
Intermediate-1	20 (44.4)
Intermediate-2	9 (20)
High	4 (8.9)
MNA (score)	25 ± 3

Mean ± SD or prevalences when appropriate. CALR, calreticulin; DIPSS, Dynamic International Prognostic Scoring System; JAK2, Janus-kinase-2; MNA, mini nutritional assessment; MPL, myeloproliferative leukemia virus oncogene.

They were compared to a healthy control group of 351 (157 males and 194 females) individuals (Table 2). The energy expenditure was measured in a subgroup of 34 patients with MF and in 63 healthy controls. The duration of follow-up at 30 October 2024 was 31 ± 8 months, and seven deaths were recorded during follow-up. The causes of death were sepsis (three cases), hemorrhagic stroke (one case), cachexia with heart failure (two cases), and cachexia with end-stage renal disease (one case). Hepatomegaly was reported in 10 participants, 3 of whom subsequently died. Portal hypertension with bleeding due to rupture of esophageal or gastric varices, or portosystemic encephalopathy did not occur in any of the participants. The two groups exhibited comparable anthropometric characteristics, but BIA reactance (*p* < 0.05) and phase angle (*p* < 0.001) values were significantly lower in the MF group (Table 2).

**Table 2:** Anthropometric, nutritional, and clinical characteristics of the group of individuals with myelofibrosis compared with a healthy control group.

	Groups		*p* ^a^
	Control	Myelofibrosis	
Sex (Male/female)	157/194	21/24	0.81
Age (years)	59 ± 9	62 ± 12	0.08
Body weight (kg)	74.2 ± 13.0	71.2 ± 13.4	0.16
Body mass index (kg/m^2^)	28.0 ± 3.8	27.1 ± 4.6	0.21
Body circumferences (cm)			
Waist	95 ± 14	95 ± 12	0.96
Hip	105 ± 7	104 ± 9	0.09
Bio-impedance analysis			
Resistance (Ω)	512 ± 82	522 ± 70	0.36
Reactance (Ω)	61 ± 10	56 ± 11	< 0.05
Phase angle (°)	6.8 ± 1.1	6.2 ± 1.1	< 0.001
Fat-free mass (kg)	53.0 ± 10.5	50.9 ± 8.4	0.15
Fat mass (%)	28.4 ± 8.1	27.6 ± 9.0	0.60
Fat-free mass index (kg/m^2^)	19.9 ± 2.5	19.4 ± 2.3	0.15
Blood pressure (mmHg)			
Systolic	128 ± 18	140 ± 22	< 0.001
Diastolic	81 ± 10	75 ± 12	< 0.005
Heart rate (beats/min)	67 ± 11	76 ± 12	< 0.001
Resting metabolic rate	31 m/32 f	16 m/18 f	
kcal/24 h	1,544 ± 327	1,654 ± 290	0.086
kcal/kg-FFM/24 h	28.5 ± 3.6	32.4 ± 4.2	< 0.001
Energy intake (kcal/day)	1,421 ± 264	1,597 ± 408	< 0.01
Habitual daily intake of			
Carbohydrates (%)	50.1 ± 10.1	44.4 ± 5.8	< 0.001
Lipids (%)	29.8 ± 6.1	33.5 ± 6.6	< 0.001
Proteins (%)	20.1 ± 2.8	22.1 ± 3.2	< 0.001
Fiber (g)	17 ± 6	18 ± 4	0.45
Glycemic index	58.0 ± 4.3	61.8 ± 8.6	< 0.005
Glucose load	105 ± 25	110 ± 29	0.22
Blood concentrations of			
Glucose (mg/dL)	90 ± 10	98 ± 13	< 0.01
Cholesterol (mg/dL)	214 ± 38	129 ± 43	< 0.001
HDL-c (mg/dL)	61 ± 16	41 ± 11	< 0.001
LDL-c (mg/dL)	132 ± 35	77 ± 31	< 0.001
Triglycerides (mg/dL)	103 ± 55	109 ± 57	0.61
Uric acid (mg/dL)	5.0 ± 1.3	5.0 ± 1.5	0.91

a Unpaired Student’s *t*-test or *χ*^2^ when appropriate.

Mean ± SD. FFM, fat-free mass; HDL-c, high-density lipoprotein cholesterol; LDL-c, low-density lipoprotein cholesterol.

**Figure 1:**
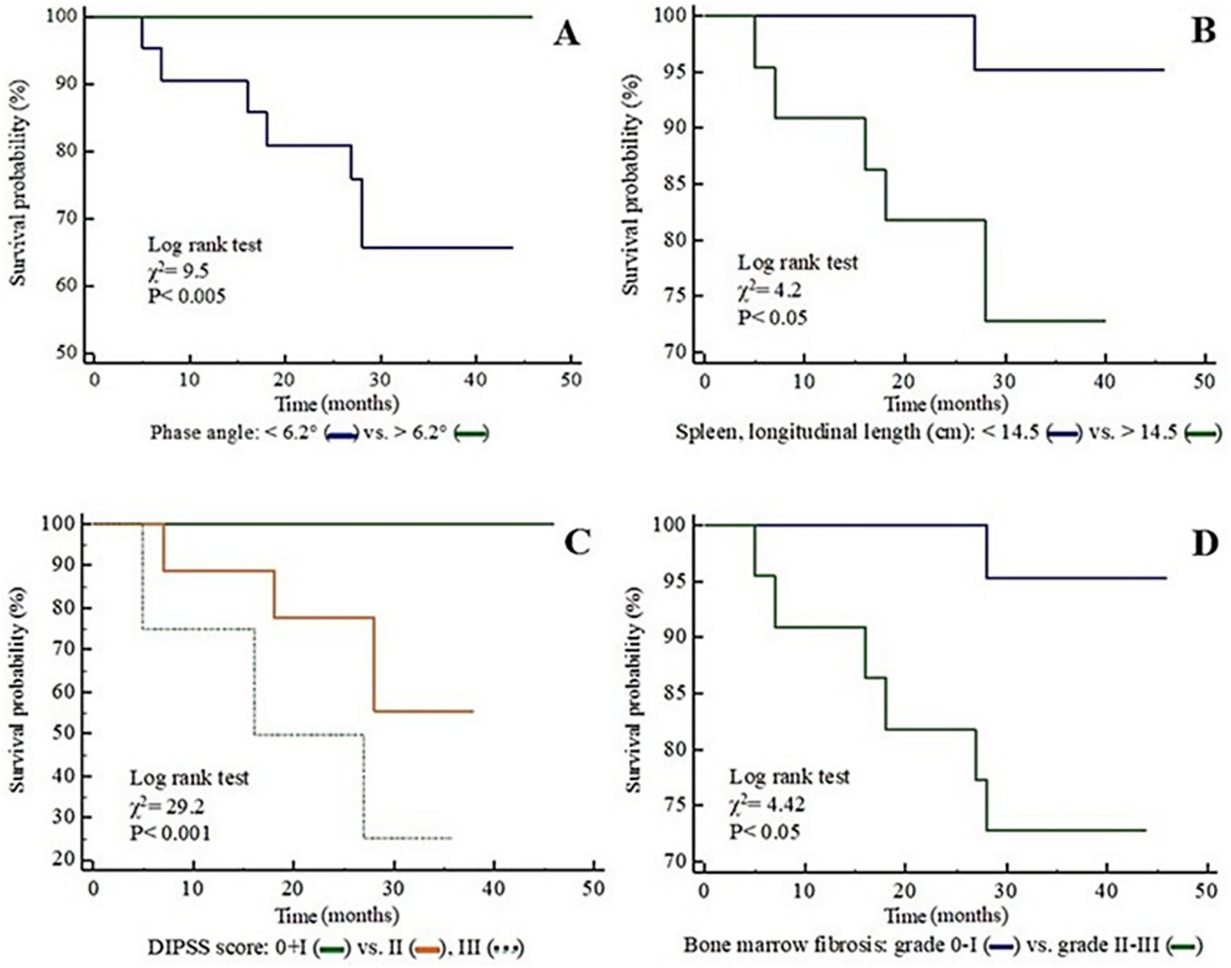
Kaplan–Meier survival curves of the patients with MF, according to phase angle **(A)**, spleen length **(B)**, DIPSS score **(C)**, and grade of bone marrow fibrosis **(D)**.

The RMR normalized for FFM was higher in the MF group (*p* < 0.001), which was also characterized by greater habitual energy intake (*p* < 0.01), with higher protein (*p* < 0.001) and fat (*p* < 0.001) consumption, but lower carbohydrate intake (*p* < 0.001) and a higher dietary glycemic index (*p* < 0.005). Compared with controls, the MF group showed higher fasting plasma glucose concentrations (*p* < 0.01) but lower total cholesterol (*p* < 0.001), HDL-cholesterol (*p* < 0.001), and LDL-cholesterol (*p* < 0.001) concentrations. In the MF group, both bioelectrical PA (*r* = −0.32; *p* < 0.05) and serum cholesterol concentrations (*r* = −0.43; *p* < 0.05) were inversely correlated with the longitudinal diameter of the spleen (Figure 1). Serum cholesterol concentrations were available in only 23 patients; therefore, these data were not included in the multivariate analysis. PA was significantly correlated with age (*r* = −0.46; *p* < 0.001), hand-grip test value (*r* = 0.47; *p* < 0.001), FFM-index (*r* = 0.43; *p* < 0.005), spleen length (*r* = −0.32; *p* < 0.05), body weight (*r* = 0.41; *p* < 0.005), and RMR (*r* = 0.18; *p* < 0.005). Multivariate analysis showed that only age, hand-grip strength, FFM-index, and spleen length were independently associated with PA (Table 3). During follow-up (18 ± 10 months), patients who died were compared with survivors (Table 4). Those who died had significantly lower reactance (*p* < 0.001) and PA (*p* < 0.001), higher RMR normalized for FFM (*p* < 0.05), and lower habitual energy intake (*p* < 0.05). They also had a higher prevalence of grade II-III bone marrow fibrosis (*p* < 0.05), longer spleen length (*p* < 0.05), greater prevalence of circulating blasts >1% (*p* < 0.001), lower hemoglobin blood concentrations (*p* < 0.05), and a higher prevalence of intermediate-2 and high DIPSS risk categories (*p* < 0.001), as well as elevated serum uric acid compared with survivors. When stratified by DIPSS risk category, patients with MF demonstrated progressively lower PA values (*p* < 0.01), increased spleen length (*p* < 0.005), and higher RMR normalized for FFM (*p* < 0.05) (Table 5).

**Table 3:** Multivariate stepwise regression analysis of factors correlated with bioelectrical phase angle.

	*ß* coefficient	Standard error	*p*
Dependent variable: phase angle			
Constant	5.46	1.32	< 0.001
Age (years)	−0.04	0.01	< 0.001
Hand-grip test (kg)	0.02	0.01	0.15
FFM index (kg/m^2^)	0.18	0.06	< 0.01
Spleen length (cm)	−0.05	0.03	0.09

Out: body weight, resting metabolic rate; F-ratio = 10,09; *p* < 0.001; *R*^2^ = 0.50. FFM, fat-free mass.

**Table 4:** Clinical and nutritional characteristics of patients with myelofibrosis according to survival at the end of follow-up.

	Patients with myelofibrosis		*p* ^a^
	Alive	Deceased	
Male/female	18/20	3/4	0.83
Age (years)	61 ± 11	72 ± 13	0.07
Diagnosis (*n*, %)			
Primary	28 (73.7)	5 (71.4)	
Secondary	10 (26.3)	2 (28.6)	0.90
Bone marrow fibrosis (*n*, %)			
Grade 0-I	22 (57.9)	1 (14.3)	
Grade II-III	16 (42.1)	6 (85.7)	< 0.05
JAK2 mutations	24 (63.2)	3 (42.9)	0.49
CALR mutations	6 (15.8)	0 (0)	0.26
MPL mutations	0 (0)	0 (0)	
Body weight (kg)	72.1 ± 13.5	65.9 ± 12.4	0.26
Body mass index (kg/m^2^)	27.2 ± 4.8	26.9 ± 3.5	0.85
Body circumferences (cm)			
Waist	96.4 ± 14.1	99.5 ± 9.0	0.45
WHR	0.92 ± 0.10	0.98 ± 0.05	< 0.05
Bio-impedance analysis			
Resistance (Ω)	526 ± 74	502 ± 44	0.27
Reactance (Ω)	59 ± 10	44 ± 6	< 0.001
Phase angle (°)	6.4 ± 1.0	5.0 ± 0.6	< 0.001
Fat-free mass (kg)	51.5 ± 8.5	47.8 ± 7.6	0.28
Fat mass (%)	27.8 ± 9.1	26.8 ± 8.1	0.77
Fat-free mass index (kg/m^2^)	19.3 ± 2.4	19.5 ± 1.2	0.81
Blood pressure (mmHg)			
Systolic	139 ± 22	146 ± 24	0.45
Diastolic	76 ± 12	71 ± 11	0.33
Heart rate (beats/min)	75 ± 12	84 ± 14	0.16
Spleen length (cm)	14.1 ± 3.5	20.7 ± 5.2	< 0.05
Time from diagnosis (months)	69 ± 50	88 ± 86	0.59
Follow-up of the study (months)	33 ± 5	18 ± 10	< 0.01
Circulatory blasts >1% (*n*, %)	3 (7.9)	6 (85.7)	< 0.001
White blood cells (× 10^9^/L)	8.90 ± 5.71	26.05 ± 21.60	0.08
Hemoglobin (g/dL)	12.9 ± 2.2	10.4 ± 2.2	< 0.05
Platelets (× 10^9^/L)	475 ± 280	548 ± 395	0.66
Absolute lymphocyte count (× 10^9^/L)	1.90 ± 1.09	3.95 ± 4.44	0.27
DIPSS risk category (*n*, %)			
Low	12 (31.6)	0 (0)	
Intermediate-1	20 (52.6)	0 (0)	
Intermediate-2	5 (13.2)	4 (57.1)	
High	1 (2.6)	3 (42.9)	< 0.001
MNA (score)	25 ± 3	24 ± 3	0.20
Resting metabolic rate			
kcal/24 h	1,656 ± 281	1,640 ± 316	0.92
kcal/kg-FFM/24 h	31.9 ± 4.0	36.0 ± 3.2	< 0.05
Energy intake (kcal/day)	1,638 ± 422	1,373 ± 224	< 0.05
Habitual daily intake of			
Carbohydrates (%)	44.8 ± 6.0	45.2 ± 5.4	0.84
Lipids (%)	33.6 ± 6.7	32.2 ± 6.1	0.48
Proteins (%)	21.6 ± 3.4	22.6 ± 2.0	0.48
Fiber (g)	18 ± 5	16 ± 3	< 0.05
Glycemic index	62 ± 9	59 ± 3	0.10
Glucose load	113 ± 30	92 ± 14	< 0.01
Blood concentrations of			
Glucose (mg/dL)	99 ± 15	98 ± 8	0.83
Cholesterol (mg/dL)	137 ± 45	107 ± 30	0.09
HDL-c (mg/dL)	44 ± 10	36 ± 14	0.19
LDL-c (mg/dL)	80 ± 30	68 ± 35	0.47
Triglycerides (mg/dL)	93 ± 33	153 ± 84	0.11
Uric acid (mg/dL)	4.5 ± 1.1	6.7 ± 1.6	< 0.05

a Unpaired Student’s *t*-test or *χ*^2^ when appropriate.

Mean ± SD. FFM, fat-free mass; HDL-c, high-density lipoprotein cholesterol; LDL-c, low-density lipoprotein cholesterol.

**Table 5:** Anthropometric, nutritional, and clinical characteristics of patients with myelofibrosis stratified according to the DIPSS risk category.

	DIPSS risk category				ANOVA
	Low	Intermediate-1	Intermediate-2	High	*p*
*n*	12	20	9	4	
BIA phase angle (°)	6.7 ± 1.2	6.4 ± 1.0	5.4 ± 0.5	5.1 ± 1.0	< 0.01
Fat-free mass index (kg/m^2^)	19.3 ± 2.3	19.5 ± 2.7	19.1 ± 1.8	19.2 ± 0.8	0.97
Energy intake (kcal/day)	1,551 ± 394	1,640 ± 423	1,604 ± 330	1,507 ± 643	0.91
Spleen longitudinal length (cm)	13.3 ± 2.6	14.0 ± 3.8	18.1 ± 4.3	20.1 ± 6.5	< 0.005
*n*	11	17	6	3	
Resting metabolic rate					
kcal/24 h	1,616 ± 340	1711 ± 244	1,591 ± 328	1,565 ± 106	0.72
kcal/kg-FFM/24 h	30.2 ± 4.2	32.8 ± 3.6	33.7 ± 3.8	38.1 ± 2.9	< 0.05

Mean ± SD. ANOVA, analysis of variance; BIA, bio-impedance analysis; DIPSS, Dynamic International Prognostic Scoring System; FFM, fat-free mass.

Kaplan–Meier survival curves (Figure 1) demonstrated that PA values below the median value were associated with a lower survival rate (*p* < 0.005), and a similar result was observed for values of spleen length above the median value (*p* < 0.05). Kaplan–Meier survival curves were significant considering the bone marrow fibrosis (grade 0-I vs. II-III; *p* < 0.05) and the DIPSS score (0 + I vs. II vs. III; *p* < 0.001). The median value of the FFM-index did not discriminate survival (*p* = 0.35), nor did it discriminate the diagnosis of primary versus secondary MF (Figure 1).

## 4 Discussion

This study investigated a heterogeneous group of individuals with MF. The results demonstrate that malnutrition is associated with MF, disease severity, and survival. Interest in malnutrition in oncology has been increasing, and in some cases, it influences survival more strongly than traditional tumor staging systems (20). However, data on hematologic malignancies, including MF remain limited (21, 22). We found that patients with MF had lower bioelectrical reactance, a marker of lean body mass, and lower PA. PA is a bioelectrical measure inversely associated with the ratio of extracellular water (ECW) to intracellular water (ICW). The ECW/ICW ratio is a well-established indicator inversely related to both nutritional and clinical wellbeing (13, 23). Consequently, low PA correlates with compromised nutritional and clinical status, where poor nutrition is often linked to poor clinical outcomes. This finding supports previous studies demonstrating that PA is a strong predictor of malnutrition and outcome across various clinical conditions (23). Multivariate analysis (Table 3) showed that PA was independently associated with age, nutritional status indicators such as FFMI and muscle strength (measured by the hand-grip test), and spleen length, which reflects disease severity (24). Kaplan–Meier survival curves further demonstrated that low PA values were significantly associated with poor survival, were as predictive as, or more predictive than, other clinical predictors such as spleen length, bone marrow fibrosis grade, and DIPSS score. Two major features of MF likely contribute to malnutrition: oncoinflammation and splenomegaly. Oncoinflammation reduces appetite and increases thermogenesis, leading to negative energy balance, loss of lean mass, and muscle wasting that may progress to cachexia (25, 26). Consistent with this mechanism, we observed that RMR normalized for FFM was higher in the MF group than in controls. Splenomegaly, through gastric compression, can induce early satiety. Thus, although the MF group demonstrated higher energy expenditure, they appeared to compensate by consuming a more energy-dense diet, with greater fat and protein intake compared with controls. Together, high energy expenditure and reduced appetite are hallmarks of cachexia. We observed that the deceased subgroup had higher energy expenditure and lower dietary energy intake, along with lower PA, compared with MF patients who survived follow-up. This suggests that, alongside worsening disease severity, such as grade II-III bone marrow fibrosis, increased circulating blast cells, and progressive splenomegaly, loss of appetite and reduced energy intake may mark the transition to cachexia and death. When the MF group was stratified by DIPSS risk category (Table 5), a progressive increase in splenomegaly was accompanied by a steady decline in PA and a rise in energy expenditure normalized for FFM.

We also found an inverse correlation between spleen length and serum cholesterol concentrations. Hypocholesterolemia may reflect malnutrition, but it has also been hypothesized that the spleen influences cholesterol metabolism, with splenomegaly contributing to lower cholesterol levels (27, 28). This observation may be relevant to the overall MF risk. However, further studies are needed to clarify the mechanisms underlying this association and its clinical significance.

This study has several limitations. First, it was a single-center study with a limited number of participants, which limited our ability to fully explore potential influences, such as pharmacological treatments on nutritional outcomes. Second, the follow-up period was relatively short and included a limited number of events. Third, bone marrow fibrosis was not documented in concomitance with nutritional measurements. In most cases, it was done at diagnosis, or it was considered the last observation before basal nutritional assessment; so, these parameters may have changed in the interim. Finally, because the design was cross-sectional and partially longitudinal observational, the associations identified cannot establish causality. Only nutritional intervention studies will clarify if improving nutritional state, regardless of MF disease severity, leads to improved patient outcomes, including quality of life. Nevertheless, a key strength of this study is the identification of nutritional predictors, specifically bioelectrical measures and energy expenditure parameters, that have not previously been examined in individuals with MF. These predictors may enhance prognostic assessment and serve as a foundation for nutritional or pharmacological interventions aimed at improving nutritional status, which could translate into better clinical outcomes.

## 5 Conclusion

Nutritional factors related to body composition and energy expenditure are important predictors of survival in individuals with MF, independent of other clinical indicators such as splenomegaly, bone marrow fibrosis, or circulating blasts. Nutritional parameters should be incorporated into supportive intervention strategies to improve both survival and quality of life of patients with MF.

## Data Availability

The datasets presented in this article are not readily available because we have no authorization from participants to share their data. Requests to access the datasets should be directed to SB, silvio.buscemi@unipa.it.
